# Neuroprotective Effect of Hydrogen-Rich Saline in Global Cerebral Ischemia/Reperfusion Rats: Up-Regulated Tregs and Down-Regulated miR-21, miR-210 and NF-κB Expression

**DOI:** 10.1007/s11064-016-1978-x

**Published:** 2016-07-07

**Authors:** Qian Li, Pan Yu, Qiuting Zeng, Bing Luo, Shenquan Cai, Kangli Hui, Gao Yu, Changsong Zhu, Xingdong Chen, Manlin Duan, Xuejun Sun

**Affiliations:** 1 Department of Anesthesia, Jinling Hospital, No. 305, Zhongshan East Road, Nanjing, 210002 Jiangsu China; 2 Department of Burn and Plastic Surgery, Jinling Hospital, No. 305, Zhongshan East Road, Nanjing, 210002 Jiangsu China; 3 Department of Anesthesia, Zhongda Hospital, Southeast University, No. 87, Hunan Road, Nanjing, 210002 Jiangsu China; 4 Department of Naval Aeromedicine, Faculty of Naval Medicine, Second Military Medical University, Shanghai, 200433 China

**Keywords:** Global cerebral ischemia/reperfusion, Neuroprotection, Hydrogen, Regulatory T cells, MiRNA, Immuno-inflammatory response

## Abstract

Recently, it has been suggested that molecular hydrogen (H_2_) can selectively reduce the levels of hydroxyl radicals (.OH), and ameliorate oxidative and inflammatory injuries to organs in global cerebral ischemia reperfusion models. Global cerebral ischemia/reperfusion (I/R) can induce a sudden activation of inflammatory cytokines and later influence the systemic immunoreactivity which may contribute to a worse outcome. Regulatory T cells (Tregs) are involved in several pathological aspects of cerebral I/R. In addition, miRNA took part in the processes of cellular response to hypoxia. Since the expression of a specific set of miRNA called “hypoxamirs” is upregulated by hypoxia. Therefore, the aim of this study was to analyze the effect of HRS on I/R inducing cerebral damage, Tregs, and specific miRNA. Our results showed that rats undergone global cerebral I/R and treated with HRS have milder injury than I/R animals without HRS treatment. miR-210 expression in the hippocampus of the I/R group at 6, 24 and 96 h after reperfusion was significantly increased at each time point, while its expression in the group treated with HRS was significantly decreased. In addition, Tregs number in group I/R was decreased at each time points, while its number in the group treated with HRS was increased at 24 and 96 h after reperfusion. We focus on the relationship among Tregs, TGF-β1, TNF-α and NF-κB at 24 h, and we found that there is a high correlation among them. Therefore, our results indicated that the brain resuscitation mechanism in the HRS-treated rats may be related with the effect of upregulating the number of Treg cells.

## Introduction

Global cerebral ischemia can cause severe central nervous system damage. However, the detailed mechanism is not fully known. Some studies suggest that activated immune cells can mediate the central nervous system injury and systemic immune inflammation, due to an early activation caused by acute cerebral ischemia and reperfusion [1]. These reactions are often accompanied by reactive oxygen species production, leading to NF-κB activation, which is involved in the activation of inflammatory gene promoters, and induction of inflammatory cytokines such as TNF-α [2]. Many studies have recently identified T lymphocytes as important mediators of ischemic brain damage, but the contribution of the different T-cell subsets is still unclear [3]. Tregs are a CD4^+^ subset, characterized by CD25^+^ expression, since immunoregulatory function is absent in CD4^+^CD25^−^ cells. Moreover, CD4^+^CD25^+^ T cells are characterized by a Forkhead box P3 (*FOXP3*) gene high expression, which is induced and sustained in Treg cells by TGF-β signaling. CD4^+^ CD25^+^FoxP3^+^ regulatory T cells (Tregs) are a component of the immune system that suppress immune responses of other cells, thus considered anti-inflammatory cells that maintain immune tolerance and counteract tissue damage in a variety of immune-mediated disorders [4]. Some studies demonstrated that Tregs infiltrate ischemic-reperfused organs during the healing process promoting repair, likely through modulation of pro-inflammatory cytokine production by other T cell subsets [5]. However, the role of Treg in the process of cerebral ischemia/reperfusion (I/R) has not been fully elucidated.

Hydrogen (H_2_), an agent that has antioxidant and anti-inflammatory properties, has been suggested as a potent free radical scavenger, since it selectively reduces the hydroxyl radical, the most cytotoxic radical among the reactive oxygen species [6, 7]. Indeed, many previous studies confirmed that hydrogen can effectively protect against tissue damage both in vivo and in vitro, including transient cerebral ischemia, neonatal cerebral hypoxia-ischemia, carbon monoxide toxicity, sepsis, traumatic brain injury, Parkinson’s disease and myocardial injury induced by I/R; however, the possible mechanism and signaling pathway is unclear [8–12].

MicroRNAs are a class of endogenous ~22 nt noncoding RNAs that can regulate gene expression at a transcriptional and/or post-transcriptional level by indirect regulation of transcription factors, post-transcriptionally by inducing mRNA degradation, or directly inhibiting the translation [13]. Several studies have showed that miRNA expression profile in cerebral ischemia-reperfusion injury is significantly changed, suggesting their implication in this disease process [14–16]. Studies over the last few years have confirmed that there is a variety of miRNA expression on T cells. These miRNAs play an important role in the regulation of development, maturation, differentiation, function and various other aspects of T cells growth. Activated CD4^+^ T cells and Treg miRNA expression profiles are very similar. The above mentioned miRNA are probably taking part in the regulation of nuclear transcription factor or other functional genes associated with activated T cells. Despite that, the miRNA specific function and role in the CD4^+^ T cells and Treg is unclear.

Numerous data suggest that hydrogen rich saline (HRS) could have a therapeutic effect on. I/R injury by involving anti-inflammatory and anti-oxidative mechanisms [9]. However, the effects of HRS on Treg and miRNA expression during I/R have not yet been reported. The present study provided evidence of HRS cerebroprotective effects in rats subjected to cerebral I/R injury, suggesting that HRS could be a novel therapeutic approach to enhance recovery from I/R injury [17]. Moreover, we confirmed that the change of Treg, NF-κB and miRNA expression was correlated with the I/R injury degree and we analyzed their correlation. We also performed tests to evaluate TGF-β1 and TNF-α concentration to confirm Treg modified content results.

## Materials and Methods

### Animals and Groups

The experimental procedures were carried out in accordance with the guidelines for the Care and Use of Laboratory Animals published by the US National Institute of Health (NIH Publication No. 85–23, revised 1996) and was approved by the Institutional Animal Care and Use Committee of Nanjing University and the Jinling Hospital, Nanjing, China. Male Sprague–Dawley rats, aged 7 weeks, weighting 280 ± 20 g, were purchased from the Experimental Animal Central of Jinling Hospital. All animals were housed under a 12 h light/dark cycle with constant temperature and free access to standard rodent chow and tap water. All rats were fasted for 12 h with water ad libitum before the operation.

In total, 123 rats were used. Three rats died during the operation. Among the 120 rats that survived, eight showed seizure after 15 min of ischemia thus, they were excluded from this study. Therefore, 112 rats were randomly divided into three groups: (1) sham group: sham operation (14 rats, T_0_); (2) sham + H_2_ group: sham operation plus HRS via intraperitoneal at the end of the operation (14 rats, T_0_, 0.16 mol/kg); (3) I/R group: 6 (14 rats, T_1_), 24 (14 rats, T_2_) and 96 h (14 rats, T_3_) ischemia reperfusion plus physiologic saline (5 ml/kg, I/R); and (4) H_2_ group: 6 (14 rats, T_1_), 24 (14 rats, T_2_) and 96 h (14 rats, T_3_) ischemia reperfusion plus HRS (0.16 mol/kg, I/R + H_2_). Either physiologic saline or HRS was intraperitoneally injected immediately after reperfusion and 6 h after reperfusion, respectively. The physiologic saline and HRS was intraperitoneally injected over 5 min [18, 19].

### Global Cerebral I/R Model

Global cerebral I/R was induced using the four-vessel occlusion (4-VO) method, as previously described [20–22]. Only rats that were unresponsive and lost the pupillary light reflexes after carotid artery occlusion were considered adequately ischemic. Animals that convulsed during and after the ischemic insult were not used in the experiments. The rectal temperature was maintained at 37 °C using a homeothermic blanket during the whole experiment. The sham-operated rats underwent anesthesia and vertebral artery occlusion without the common carotid arteries occlusion.

### HRS Preparation

HRS was prepared as previously described [23] using an equipment provided by the Department of Diving Medicine, the Second Military Medical University, China. HRS was stored under atmospheric pressure at 4 °C in an aluminum bag with no dead volume and was sterilized by gamma radiation. Gas chromatography was used to confirm the hydrogen level content in the saline by the method described by Ohsawa et al. [24]. HRS was freshly prepared every week to ensure that the hydrogen concentration of more than 0.8 mmol/L was maintained.

### Behavioral Test

To investigate the neurobehavioral manifestations after global cerebral I/R, Neurological Severity Scores (NSS) were evaluated in all the rats of each group at T_0_, T_1_, T_2_ and T_3_. The test was performed by inspectors blinded to the groups. According to the NSS, the neurological function was evaluated on a scale of 0–18: *movement/activity* (no spontaneous movement = 0; sluggish movement = 1; normal movement = 2); *respirations* (irregular breathing pattern = 0; decreased breathing frequency with normal pattern = 1; normal breathing frequency and pattern = 2); *consciousness* (no reaction to pinching of tail = 0; poor response to tail pinch = 1; normal response to tail pinch = 2); *coordination* (failure in 1 cm wide beam balancing task and balances with steady posture, paws on top of beam = 1; grasps sides of beam and/or has shaky movement = 1; one or more paws slip off beam = 1; attempt to balance on beam but falls off = 1; drapes over beam and/or hangs on beam and falls off = 1; falls off beam with no attempt to balance or hang on = 1); and *corneal reflex* (no blinking = 0; sluggish blinking = 1; normal blinking = 2). A score of 13–18 points indicates severe injury; 7–12 moderate injury; and 1–6 mild injury [25, 26].

### Preparation of Brain Slices in the CA1 Area of the Hippocampus

The brain tissues of three rats in each group were harvested at T_0_, T_1_, T_2_ and T_3_. The rats were anesthetized with 2 % pentobarbital (0.3 ml/100 g body weight, i.p.), perfused through the systemic circulation with cold 0.9 % saline (4 °C) until the backflow fluid in the right atrium was clear, and their eyes and four paws were pale, then perfused through systemic circulation again with 200 ml 4 % paraformaldehyde (PBS 0.1 M, pH 7.4). Then the cerebral cavity was carefully opened and the brain was quickly removed. Approximately 6-mm thick coronal brain tissues were taken with up to 1–2 mm of cerebellum. The coronal brain tissue was immersed in 4 % paraformaldehyde (PBS 0.1 M, pH 7.4) and stored at 4 °C for 24 h. Coronal brain slices containing hippocampus of about 5-mm thick were cut and embedded in paraffin. The brain slices were sequentially cut into 4-μm thick sections for further immunohistochemical staining and hematoxylin and eosin staining (HE).

### Pyramidal Cell Morphology and Cell Count Detection

Half of the aforementioned 4-μm thick sections were stained with HE for light microscope observation. The CA1 area of the hippocampus from each animal was captured and Imaging-Pro-Plus (Leica DMLB, Solms, Germany) was used to perform cell number quantitative analysis. Histological changes in the CA_1_ hippocampus sections were evaluated by a blind rater under an optical microscope. Four fields (×400) were sequentially selected for each section, and the numbers of pyramidal cells present inside were counted. Normal pyramidal cells have relatively big cell body, rich in cytoplasm, with one or two big round nuclei. In contrast, damaged cells show shrunken cell body, condensed nuclei, dark cytoplasm, and many empty vesicles.

### Immunohistochemical Staining

The remaining half of the 4-μm thick sections underwent standard immunohistochemical staining. The slices were incubated with 3 % H_2_O_2_, 3 % normal goat serum and incubated with primary antibodies in 0.01 mol/l phosphate-buffered saline at 4 °C overnight. Rabbit anti-rat NF-κB p65 antibody (1:50, Cell Signaling Technology, USA), and rabbit anti-rat IgG-HRP antibody (1:100, Cell Signaling Technology, USA) were used. Immunohistochemistry was performed via the avidin biotin technique, and hematoxylin was selected as counterstaining. The secondary antibodies, secondary biotinylated conjugates and diaminobenzidine were from the SP rabbit kit (Cowin Biology Technology Company, China). An examiner blinded to the experimental groups counted the NF-κB positive cells under a BX53 light microscope (Olympus, Japan). For each sample, four fields (×400) were sequentially selected for each section, and the numbers of positive cells were counted. The average positive cells of each slice were obtained by dividing the sum of the positive cells counted from each field by four. The average of each sample was used for statistical analysis.

### Preparation of Hippocampus Tissues

At 24 h reperfusion, three rats were anesthetized (10 % chloral hydrate, 400 mg/kg, i.p), and systemic circulation was perfused with 0.9 % saline on ice until their eyes and paws were pale. The rats cerebral cavity was carefully opened, and the hippocampus was immediately removed. Hippocampus tissue was homogenized with 9 volumes of 4 °C saline into 10 % tissue homogenate and centrifuged at 3000×*g* for 20 min. The supernatant was collected for TNF-α detection.

### Total RNA Isolation and Real-Time RT-PCR

Four rats in each group were used for miRNA analysis. The hippocampus was lysed and total mRNA was isolated using the Trizol reagent, according to the manufacturer’s protocol (Life Technologies Corporation, Netherlands). Total mRNA was reverse-transcribed into cDNA using RevertAid first Strand cDNA Synthesis Kit (Invitrogen, Frederick, MD, USA) for Quantitative PCR (TaKaRa Bio Group, Japan) in the presence of a fluorescent dye (SYBR Green I, Cwbio). miR-210 and miR-21 were reverse-transcribed and PCR primers were designed in reference to the method of Chen et al. [27, 28]. U-87 was used as an internal reference. RT-qPCR was used to analyze miR-210 and miR-21 expressions at 6, 24 and 96 h. Primer sequences were as follows:

- *miR-210* Rt-PCR primer: GTCGTATCCAGTGCAGGGTCCGAGGTATTCGCACTGGATACGACTCAGCC; upstream primer: AGCGTGCTGTGCGTGTGAC; downstream primer: CAGTGCAGGGTCCGAGGTATT. The length of amplified products was 64 bp.
- *miR-21* Rt-PCR primer: GTCGTATCCAGTGCAGGGTCCGAGGTATTCGCACTGGATACGACTCAACA; ipstream primer: GCCGCGTAGCTTATCAGACT; downstream primer: CAGTGCAGGGTCCGAGGTATT. The length of amplified products was 61 bp.
- *U87* Upstream primer: ACAATGATGACTTATGTTTTT; downstream primer: GCTCAGTCTTAAGATTCTCT. The length of amplified products was 72 bp.

### Flow Cytometry Analysis

Blood samples of four rats were collected at T_0_, T_1_, T_2_ and T_3_ for cell population analysis by flow cytometry. Venous blood (approximately 50 μl) was harvested from the eyes of anesthetized rats and collected in heparinized tubes. All blood samples were washed once in phosphate-buffered saline (PBS) containing 1 % fetal calf serum (FCS) and stained with the following combination of fluorescently labeled anti-mouse monoclonal antibodies (eBioscience, San Diego, CA, USA): anti-CD4 and anti-CD25 for 15 min. After surface staining, the samples were permeabilized by incubation with the fixation/permeabilization buffer (eBioscience, San Diego, CA, USA) and incubated with anti-Foxp3 monoclonal antibody (mAbs) for 30 min. After incubation with anti-Foxp3, cells were analyzed by flow cytometry using a Becton Dickinson (BD) FACS Caliber and Cell Quest Software (BD Biosciences, San Jose, CA, USA). Isotype-matched antibodies were used as controls for all samples.

### Splenocyte Culture In Vitro

Spleens were harvested simultaneously with blood samples under sterile conditions. A single-cell suspension was prepared by passing the tissue through a 200 µm nylon mesh screen. The filtrate was washed using RPMI 1640 (Gibco-Invitrogen, Carlsbad, CA, USA) and then centrifuged to remove the supernatant. Red cells in the centrifuged sample were lysed using red cell lysis buffer and the supernatant obtained by centrifugation (1200 rev/min) was removed. Red blood cells were removed from splenocytes then washed with RPMI 1640, counted and adjusted to 5 × 10^6^ cells/ml in RPMI medium, which we called spleen blood mononuclear cell (SBMC) [29]. SBMC were filtered in a sterile nylon column and the opaque filtrate was harvested in culture plates. The culture plates were incubated for 72 h at 37 °C and 5 % CO_2_. After 3 days, the supernatants were harvested and stored at −80 °C for cytokines detection by ELISA assay.

### Enzyme-Linked Immunosorbent Assay

Quantitative TGF-β1 level determination in the spleen and TNF-α in the hippocampus were evaluated using a standard ELISA according to the manufacturer’s instructions (ELISA Set, R&D Systems). Values were expressed in pg/ml. Optical density was measured at 450 nm within 10 min and protein concentrations were determined using a provided standard curve.

### Statistical Analysis

Data were expressed as the mean ± SD. Statistical analysis was carried out using the *SPSS Statistics 13.0* (SPSS Inc., Chicago, IL, USA). Differences between multiple groups were analyzed by the one-way analysis of variance (ANOVA). Changes between experimental groups were determined by the Dunnett’s T3 post hoc test. Bivariate analysis was performed to evaluate the reliability of miR-210, miR-21, Treg, TGF-β1, NF-κB, TNF-α and the cerebral ischemia-reperfusion injury severity; the correlation coefficient was r. A value of *P* < 0.05 was considered statistically significant.

## Results

### HRS Reduced Global Cerebral I/R Damage

**Fig. 1:**
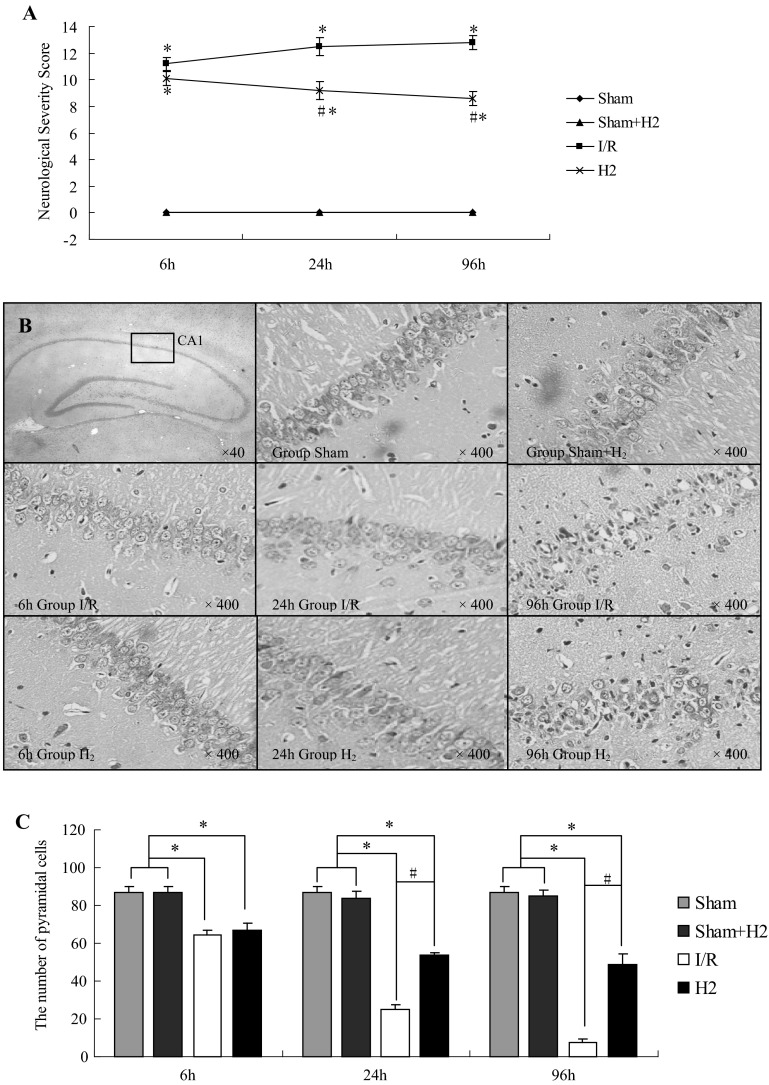
Hydrogen-rich saline (HRS) reduces brain injury after global cerebral I/R in rats. **a** NSS is increased at the three time points after I/R vs the Sham and Sham + H_2_ groups. HRS decreased brain injury at 24 and 96 h. *P < 0.05 vs sham and Sham + H_2_ groups, ^#^P < 0.05 vs I/R group. Values are expressed as mean ± SD, n = 14. **b** Pyramidal cells in the CA_1_ area of rat hippocampus from the four treatment groups at different time points. **c** The number of pyramidal cells in the CA1 area of the hippocampus at 6, 24, and 96 h after I/R with or without HRS; *P < 0.05 vs Sham and Sham + H_2_ groups, ^#^P < 0.05 vs I/R group. Values are expressed as mean ± SD, n = 3

To investigate the effect of HRS on the global cerebral I/R in rats, behavioral test at T_1_, T_2_ and T_3_, qualitative and quantitative analysis of pyramidal cell were performed at T_0_, T_1_, T_2_ and T_3_. Compared with the sham and sham + H_2_ groups, NSS increased in both I/R and H_2_ groups at each time point after reperfusion. However, the NSS in the I/R group was higher than H_2_ group at T_2_ and T_3_ (*P* < 0.05) (Fig. 1a). Indeed, the I/R group showed a time-dependent NSS increase, while the H_2_ group showed a NSS increase at T1 followed by a decrease at T2 and T3 (*P* < 0.05).

The pyramidal cells in the CA_1_ area of the hippocampus of the rats belonging to the sham and sham + H_2_ groups were close to each other and neatly arranged. Compared with the sham and sham + H_2_ groups, the cells in the CA_1_ area of the hippocampus of the I/R group revealed occasional nuclear atypia at 6 h after reperfusion although the cell arrangement was still the usual one of the controls. However, at 24 h after reperfusion the pyramidal cells were loosely arranged and the number of dead cells was increased. At 96 h after reperfusion the pyramidal cells showed a large number of intracellular vacuoles and they are sparsely arranged, their nuclei were condensed. On the other hand, the cells in the CA_1_ area of the hippocampus of the H_2_ group were closely and neatly arranged at 6 h after reperfusion. At 24 h after reperfusion, sporadic pyramidal cell death was observed while the arrangement was still the usual one of the controls. At 96 h after reperfusion some pyramidal cells showed karyopyknosis and cell death was increased, although a normal arrangement of the pyramidal cells was still visible and the cell damage was less prominent than the I/R group at 96 h (Fig. 1b).

Compared with the sham and sham + H_2_ groups, the number of pyramidal cells in the CA_1_ area of the hippocampus at 6, 24 and 96 h after reperfusion in both the I/R and H_2_ group was significantly decreased (*P* < 0.05) in a time-dependent manner. On the other hand, the cell number in group H_2_ was significantly higher at 24 and 96 h after reperfusion compared with the I/R group (*P* < 0.05) (Fig. 1c).

### HRS Regulated miR-210 and miR-21 in the Hippocampus After I/R

**Fig. 2:**
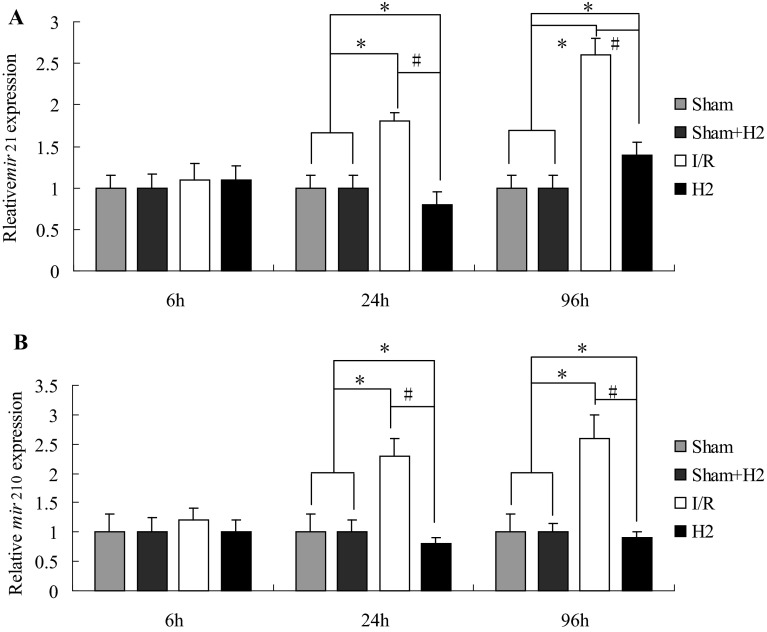
MicroRNA-21 and MicroRNA-210 expression are altered in the hippocampus after I/R. **a** miR-21 expression by qRT-PCR analysis of total RNA isolated from hippocampal tissue harvested at the indicated times after injury. miR-21 expression in the hippocampus at 6, 24, and 96 h after I/R with or without HRS; *P < 0.05 vs Sham and Sham + H_2_ groups, ^#^P < 0.05 vs I/R group. Values are mean ± SD, n = 4. **b** miR-210 expression by qRT-PCR analysis in the hippocampus at the indicated times after injury. *P < 0.05 vs Sham and Sham + H_2_ groups, ^#^P < 0.05 vs I/R group. Values are expressed as mean ± SD, n = 4

Since increasing evidence suggests that miRNA expression profile in cerebral I/R injury changes significantly, miR-210 and miR-21 in the hippocampus were measured at 6, 24 and 96 h after reperfusion. Compared with the sham and sham + H_2_ groups, miR-210 and miR-21 expression in group I/R was significantly increased at 24 and 96 h after reperfusion (*P* < 0.05), while miR-210 and miR-21 expression in group H_2_ was significantly decreased at 24 and 96 h compared with group I/R (*P* < 0.05) (Fig. 2). The expression of miR-210 and miR-21 in all groups was negatively correlated with the pyramidal cells number in the CA_1_ area of the hippocampus at 24 h after reperfusion (*P* < 0.05, Pearson correlation coefficient *r* = −0.65 and *P* < 0.05, *r* = −0.84, respectively).

### HRS Influenced Immune System Response After I/R

**Fig. 3:**
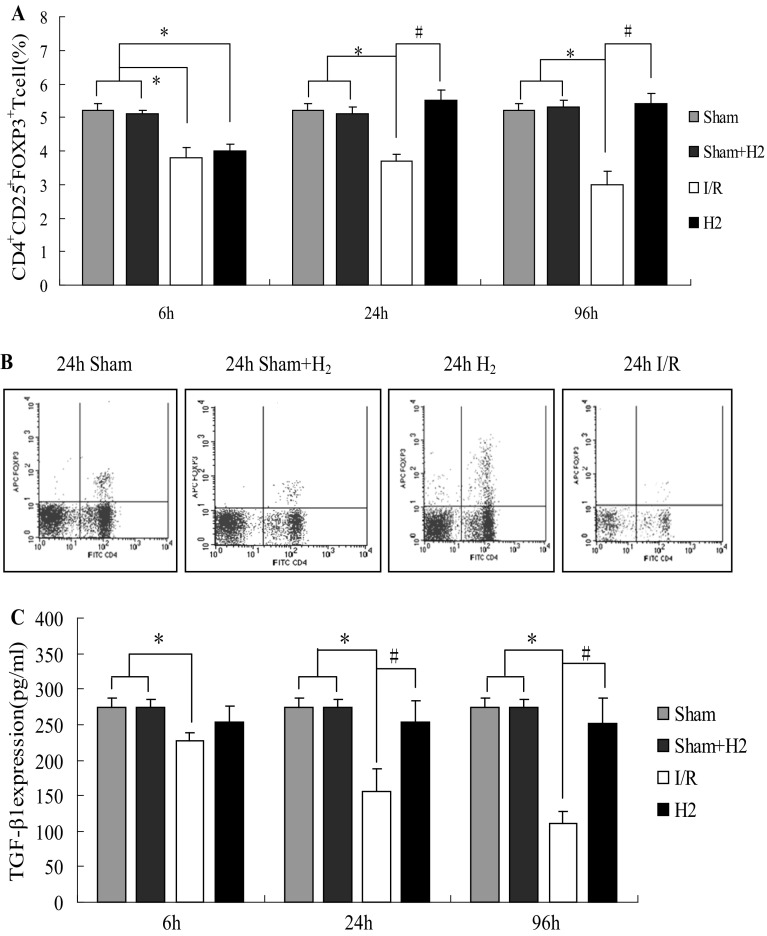
Regulatory T cells in the periphery and TGF-β1 spleen level in each group were respectively evaluated by flow cytometry and ELISA. **a** Treg cell numbers at 6, 24, and 96 h after I/R with or without HRS; *P < 0.05 vs Sham and Sham + H_2_ groups, ^#^P < 0.05 vs I/R group. Values are mean ± SD, n = 4. **b** Flow cytometry assay at 24 h after I/R. **c** TGF-β1 in the spleen at 6, 24, and 96 h after I/R with or without HRS; *P < 0.05 vs Sham and Sham + H_2_ groups, ^#^P < 0.05 vs I/R group. Values are expressed as mean ± SD, n = 4

**Fig. 4:**
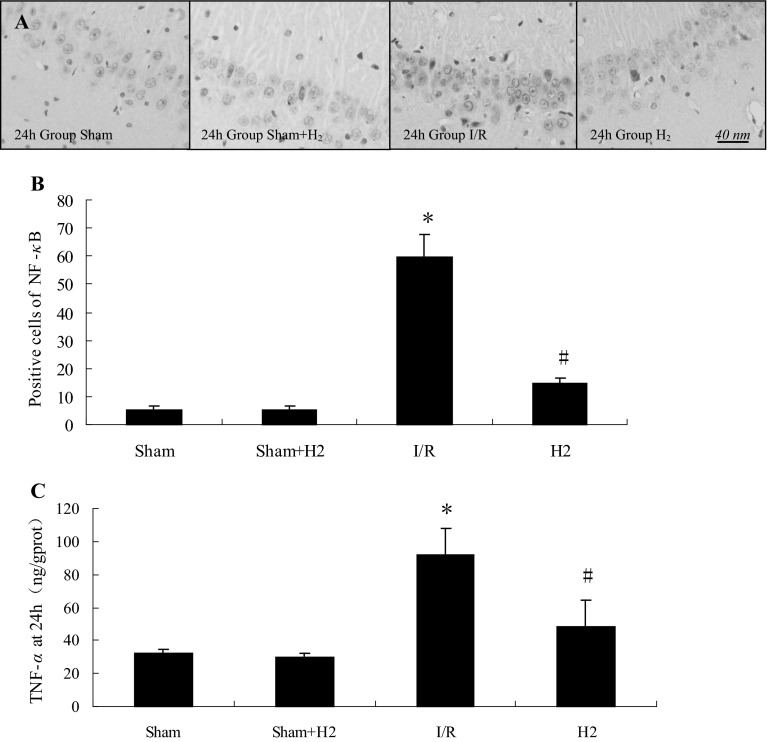
NF-κB immunohistochemical staining and positive cell counts, and TNF-α concentration in the hippocampus. **a** NF-κB immunohistochemical staining in the hippocampus in rats at 24 h after I/R. **b** NF-κB positive cell counts in the CA1 area of the hippocampus at 24 h after reperfusion. *P < 0.05 vs Sham and Sham + H_2_ groups, ^#^P < 0.05 vs I/R group. Values are mean ± SD, n = 3. **c** TNF-α concentration in the hippocampus at 24 h after reperfusion. *P < 0.05 vs sham group, ^#^P < 0.05 vs I/R group. Values are expressed as mean ± SD, n = 3

To determine whether HRS could influence the immune system response after global cerebral I/R in rats, regulatory T cells in the periphery and the concentration of spleen TGF-β1 from each group were evaluated at 6, 24 and 96 h by flow cytometry and ELISA. Compared with the sham and sham + H_2_ groups, the Treg cell population was decreased in the I/R group at T_1_,T_2_ and T_3_ time points (*P* < 0.05). The Treg cell population was decreased in the H_2_ group at T_1_ time point (*P* < 0.05), compared with group I/R Treg cell population that was increased at T_2_ and T_3_ time points in group H_2_ (*P* < 0.05) (Fig. 3a, b). TGF-β1 expression was decreased in the I/R group at T_1_,T_2_ and T_3_ time points compared with the sham and sham + H_2_ groups (*P* < 0.05). TGF-β1 expression was increased at T_2_ and T_3_ time points in the H_2_ group compared with the I/R group (*P* < 0.05) (Fig. 3c). Treg were positively correlated with TGF-β1 expression in all the groups (*P* < 0.05, *r* = 0.607). NF-κB expression in the hippocampus (an important pro-inflammatory mediator), and TNF-α concentrations in the hippocampus from each group were evaluated at 24 h by immunohistochemical staining and ELISA. NF-κB positive cell numbers in the CA1 area of the hippocampus at 24 h in the H_2_ group were higher than the sham and sham + H_2_ groups, but significantly lower than the I/R group (*P* < 0.05, Fig. 4a, b). TNF-α concentrations at 24 h in the hippocampus of the H_2_ group were also higher than in the sham and sham + H_2_ groups but significantly lower than in the I/R group (*P* < 0.05, Fig. 4c).

## Discussion

### HRS Reduced I/R Brain Injury

Hydrogen is a potent free radical scavenger that is thought to induce protective effects through anti-oxidative stress and apoptotic pathways. Our previous studies demonstrated that hydrogen (H_2_) reduced radiation-induced caspase-3 and Bax activation, and enhanced bcl-2 levels after cerebral I/R [10, 30]. We also demonstrated that intraperitoneal injection of HRS at 6 h after cerebral I/R was more efficient than an intraperitoneal injection during reperfusion. Some researchers have found that immediately after cerebral ischemia and reperfusion, cellular oxidative phosphorylation capacity was reduced, inflammatory cells were activated, large amounts of inflammatory cytokines and reactive oxygen species were released, then inflammatory cytokines in the brain tissue peaked at 6 h after reperfusion [31, 32]. In this study, we choose to perform a HRS intraperitoneal injection immediately after reperfusion and 6 h after reperfusion to treat cerebral I/R injury in rats.

Cerebral I/R can cause serious neuronal injury and death, which can further lead to learning and memory impairment and neurodegeneration. The pyramidal neurons in the CA1 area of the hippocampus are essential for spatial learning and memory functions. When suffering from cerebral ischemia insult, the hippocampal pyramidal neurons are the most vulnerable to the reduction of blood supply to the brain, and cell death occurs days after the initial ischemic insult, a phenomenon termed “delayed neuronal death” [33]. Currently, the mechanisms of neuronal injury and death induced by cerebral I/R are not completely known, and therefore an effective therapy for ischemic cerebral damage has remained elusive. In this study, HRS significantly increased the number of surviving cells in the rats I/R CA1 area of the hippocampus compared with the I/R group. Similarly, the test of neurobehavioral test confirmed that HRS reduced I/R brain injury. This protective effect could be due to the diffusion rate of HRS. Indeed, hydrogen has a good diffusion rate, can easily penetrate the blood–brain barrier, and reach the deep brain tissue, and it is also able to reach the site of injury before revascularization to remove toxic oxygen free radicals [34].

### HRS Modulated miR-210 and miR-21 Expression

Ischemia disrupts the balance between endogenous oxidants and antioxidants and overproduces toxic free radicals, thus plenty of ROS are generated during an acute ischemic stroke. Reperfusion also comes with massive production of reactive oxygen species (ROS) and reactive nitrogen species (RNS) that potentiate initial brain injury. Hydroxyl radicals easily react with DNA, proteins and lipids, to evoke disastrous cytotoxic effect [32].

Studies have reported that miRNA is involved in the process of cellular responses to hypoxia. In recent years, many independent studies have demonstrated that the expression of a specific set of miRNA is upregulated by hypoxia. These uniquely induced miRNA were termed as “hypoxamirs”, and they appear to control a network of multiple processes during the hypoxic response [35]. Many studies have identified miRNA-210 (miR-210) as a unique hypoxamirs that is robustly and ubiquitously induced in both transformed and primary cell types [36, 37]. MiR-21 is an effective anti-apoptotic factor and its main function is to act on a specified pro-apoptotic gene. Aberrant miR-21 expression has been reported in traumatic brain injury and increased miR-21 expression has been linked to increased cell survival, growth, and proliferation and decreased apoptosis [38, 39]. Our data showed that miR-210 expression was up-regulated after 15 min ischemia and 6 h reperfusion, and the elevated miR-210 expression peaked at 96 h post-injury. The trend of miR-21 expression was similar to that of miR-210. When the cerebral I/R injury was treated with HRS immediately after reperfusion, miR-210 and miR-21 expressions were decreased significantly at 24 and 96 h. Our results of an increased miR-210 and miR-21 after I/R injury and their decrease in the HRS treated rats, together with the histological examination and behavioral test results shown in our study, suggested that miR-210 and miR-21 may be considered markers of cerebral I/R injury.

### HRS Regulated Immune System Response After I/R

Originally thought to be completely separated from the peripheral immune system, it is now recognized that activated immune cells can access the brain without requiring local trauma [25]. The mechanisms that regulate the immunity response after cerebral ischemia are multiple. An important component of the mechanisms is the overactive immune responses that can lead to CNS damage with wide and far-reaching consequences to survival [40]. Therefore, it is vitally important to maintain the immune balance to reduce brain damage. The spleen is an important immune organ that consists of aggregates of lymphoid tissue [41], and therefore was chosen for analysis of representative immunological functions in this study. Regulatory T cells (Tregs) are antigen specific, possessing T-cell receptors, and they possess a suppressive function. Indeed, they modulate the immune system, maintain tolerance to self-antigens, abrogate autoimmune disease and suppress immune responses of other cells [42]. This is an important “self-check” built into the immune system to prevent excessive reactions. In rats and human, Treg cells are mainly distributed in the peripheral blood and spleen, with a percentage of approximately 5–10 % peripheral CD4^+^T cells [43]. In this experiment, Treg cells number in the rats after I/R decreased significantly at different time points. It is noteworthy that Treg cells number continuously declined in the I/R group while markedly increased in the rats treated with HRS at 24 h after reperfusion. We hypothesized that at reperfusion 24 h, the vivo immune suppression on the rise in rats treated with HRS. The suppression of an overactive immune response can reduce CNS damage.

TGF-β_1_ is an important immunomodulatory cytokine, which has a strong immunosuppressive activity. Experiments in vivo and in vitro confirmed that TGF-β_1_ can significantly reduce nerve cells death and palliate inflammatory injury after cerebral ischemia. Treg cells can secrete TGF-β_1_ while TGF-β_1_ can down-regulate Treg cells to suppress T cell responses and maintain immune tolerance [44]. In this experiment, the number of Treg cells and TGF-β1 expression were positively correlated, which also confirmed the relationship between Treg and TGF-β1.

Ischemia/reperfusion disrupts the balance between systemic immunity and reactive inflammatory cytokines, thus free radicals/oxidants released by inflammatory cells can severely threaten tissue viability in the brain. Inflammatory cytokines play important roles in the neuropathology of brain I/R injury, and the reduction of inflammation was correlated with neuroprotective effects [45]. A study by Tian et al., reported that HRS treatment of TBI (traumatic brain injury)-challenged rats decreased the levels of pro-inflammatory cytokines (TNF-α, IL-1β and HMGB1), inflammatory cell numbers (Iba1) and inflammatory metabolites (Cho) and increased the levels of an anti-inflammatory cytokine (IL-10) in the brain tissues of TBI-challenged rats [18]. In this study, NF-κB in the hippocampus as an important pro-inflammatory mediator and TNF-α concentration in the hippocampus were evaluated. During reperfusion, the production of reactive oxygen species increased over a short period, activating NF-κB. Once activated, NF-κB combines with the gene promoters that are involved in inflammation, leading to the production of inflammatory cytokines, among which the most representative is TNF-α [46]. We found NF-κB expression and TNF-α concentrations in the hippocampus were significantly reduced by HRS in I/R rats at 24 h after reperfusion. Simultaneously, the number of Treg cells, as important anti-inflammatory mediators, and TGF-β1 concentration, as an anti-inflammatory cytokine, markedly increased in the rats treated with HRS compared with the I/R group. However, there may be many interactions between the central immunity and the peripheral immunity. HRS suppressive effect on immunity might be related to an immunosuppression effect. The exact mechanism still needs further studies.

Recently, it has been reported that more miRNAs are involved in the regulation of the immune system, demonstrating that miRNAs modulate many aspects of the immune responses such as differentiation, proliferation, cell fate determination, function of immune cells, and cytokine responses, as well as the intracellular signaling pathways [47]. Some miRNAs have the potential to broadly influence the molecular pathways that control the development and function of innate and adaptive immune responses. Some study found that miR-210 was down-regulated in Tregs compared with controls and it has potential target sites in the 3-UTR of FOXP3. FOXP3 expression is negatively regulated by miR-210 direct binding to their two target sites in its 3-UTR [48]. Therefore, further in-depth study about miRNAs and Tregs will be performed.

In summary, the present work provided some evidence in support of the hypothesis that HRS could offer a neuroprotective effect on acute global cerebral I/R damage. In particular, HRS beneficial effects against I/R injury were likely exerted by Treg, TGF-β1 up-regulation and miR-21, miR-210, NF-κB and TNF-α down-regulation. Therefore, HRS provided an effective pharmacological effect of potential therapeutic applications for future treatment of acute global cerebral ischemia and reperfusion.

## References

[1] Hotchkiss RS, Strasser A, McDunn JE, Swanson PE (2009). Cell death. N Engl J Med.

[2] Lu L, Tang D, Wang L, Huang LQ, Jiang GS, Xiao XY, Zeng FQ (2012). Gambogic acid inhibits tnf-alpha-induced invasion of human prostate cancer pc3 cells in vitro through pi3k/akt and nf-kappab signaling pathways. Acta Pharmacol Sin.

[3] Huang Y, Rabb H, Womer KL (2007). Ischemia-reperfusion and immediate T cell responses. Cell Immunol.

[4] Aggarwal BB (2010). Targeting inflammation-induced obesity and metabolic diseases by curcumin and other nutraceuticals. Annu Rev Nutr.

[5] Xu WH, Zhang AM, Ren MS, Zhang XD, Wang F, Xu XC, Li Q, Wang J, Din BS, Wu YB, Ghet Chen (2012). Changes of Treg-associated molecules on CD4+ CD25+ Treg cells in myasthenia gravis and effects of immunosuppressants. J Clin Immunol.

[6] Hou Z, Luo W, Sun X, Hao S, Zhang Y, Xu F, Wang Z, Liu B (2012). Hydrogen-rich saline protects against oxidative damage and cognitive deficits after mild traumatic brain injury. Brain Res Bull.

[7] Ji X, Tian Y, Xie K, Liu W, Qu Y, Fei Z (2012). Protective effects of hydrogen-rich saline in a rat model of traumatic brain injury via reducing oxidative stress. J Surg Res.

[8] Mano Y, Kotani T, Ito M, Nagai T, Ichinohashi Y, Yamada K, Ohno K, Kikkawa F, Toyokuni S (2014). Maternal molecular hydrogen administration ameliorates rat fetal hippocampal damage caused by in utero ischemia-reperfusion. Free Radic Biol Med.

[9] Hayashida K, Sano M, Ohsawa I, Shinmura K, Tamaki K, Kimura K, Endo J, Katayama T, Kawamura A, Kohsaka S, Makino S, Ohta S, Ogawa S, Fukuda K (2008). Inhalation of hydrogen gas reduces infarct size in the rat model of myocardial ischemia-reperfusion injury. Biochem Biophys Res Commun.

[10] Ji Q, Hui K, Zhang L, Sun X, Li W, Duan ML (2011). The effect of hydrogen-rich saline on the brain of rats with transient ischemia. J Surg Res.

[11] Hayashida K, Sano M, Kamimura N, Yokota T, Suzuki M, Ohta S, Fukuda K, Hori S (2014). Hydrogen inhalation during normoxic resuscitation improves neurological outcome in a rat model of cardiac arrest independently of targeted temperature management. Circulation.

[12] Fu Y, Ito M, Fujita Y, Ito M, Ichihara M, Masuda A, Suzuki Y, Maesawa S, KajitaY, Hirayama M, Ohsawa I, Ohta S, Ohno K (2009). Molecular hydrogen is protective against 6-hydroxydopamine-induced nigrostriatal degeneration in a rat model of Parkinson’s disease. Neurosci Lett.

[13] Sun H, Chen L, Zhou W, Hu L, Li L, Tu Q, Chang Y, Liu Q, Sun X, Wu M, Wang H (2011). The protective role of hydrogen-rich saline in experimental liver injury in mice. J Hepatol.

[14] Quero L, Dubois L, Lieuwes NG, Hennequin C, Lambin P (2011). miR-210 as a marker of chronic hypoxia, but not a therapeutic target in prostate cancer. Radiother Oncol.

[15] Jeyaseelan K, Lim KY, Armugam A (2008). MicroRNA expression in the blood and brain of rats subjected to transient focal ischemia by middle cerebral artery occlusion. Stroke.

[16] Truettner JS, Alonso OF, Bramlett HM, Dietrich WD (2011). Therapeutic hypothermia alters microRNA responses to traumatic brain injury in rats. J Cereb Blood Flow Metab.

[17] Ji Q, Hui K, Zhang L, Sun X, Li W, Duan M (2011). The effect of hydrogen-rich saline on the brain of rats with transient ischemia. J Surg Res.

[18] Tian R, Hou Z, Hao S, Wu W, Mao X, Tao X, Lu T, Liu B (2016). Hydrogen-rich water attenuates brain damage and inflammation after traumatic brain injury in rats. Brain Res.

[19] Han L, Tian R, Yan H, Pei L, Hou Z, Hao S, Li YV, Tian Q, Liu B, Zhang Q (2015). Hydrogen-rich water protects against ischemic brain injury in rats by regulating calcium buffering proteins. Brain Res.

[20] Pulsinelli WA, Buchan AM (1988). The four vessel occlusion rat model method for complete occlusion of vertebral arteries and control of collateral circulation. Stroke.

[21] Toda S, Ikeda Y, Teramoto A, Hirakawa K, Uekusa K (2002). Highly reproducible rat model of reversible forebrain ischemia—modified four-vessel occlusion model and its metabolic feature. Acta Neurochir.

[22] Liu J, Liu WB, Ji XT, Fei Z, Cheng G (2012). One-stage apertura thoracis superior approach for four-vessel occlusion in rats. Chin J Traumatol.

[23] Cai J, Kang Z, Liu K, Liu W, Li R, Zhang JH, Luo X, Sun X (2009). Neuroprotective effects of hydrogen saline in neonatal hypoxia-ischemia rat model. Brain Res.

[24] Ohsawa I, Ishikawa M, Takahashi K, Watanabe M, Nishimaki K, Yamagata K, Katsura K, Katayama Y, Asoh S, Ohta S (2007). Hydrogen acts as a therapeutic antioxidant by selectively reducing cytotoxic oxygen radicals. Nat Med.

[25] Chang L, Chen Y, Li J, Liu Z, Wang Z, Chen J, Cao W, Xu Y (2011). Cocaine-and amphetamine-regulated transcript modulates peripheral immunity and protects against brain injury in experimental stroke. Brain Behav Immun.

[26] Albertsmeier M, Teschendorf P, Popp E, Galmbacher R, Vogel P, Bottiger BW (2007). Evaluation of a tape removal test to assess neurological deficit after cardiac arrest in rats. Resuscitation.

[27] Chen H, Sun YP, Li Y, Liu WW, Xiang HG, Fan LY, Sun Q, Xu XY, Cai JM, Ruan CP, Su N, Yan RL, Sun XJ, Wang Q (2010). Hydrogen-rich saline ameliorates the severity of l-arginine-induced acute pancreatitis in rats. Biochem Biophys Res Commun.

[28] Singhal AB, Lo EH (2008). Advances in emerging nondrug therapies for acute stroke. Stroke.

[29] Bik W, Skwarlo-Sonta K, Szelagiewicz J, Wolinska-Witort E, Chmielowska M, Martynska L, Baranowska-Bik A, Baranowska B (2008). Involvement of the cocaine-amphetamine regulated transcript peptide (CART55-102) in the modulation of rat immune cell activity. Neuro Endocrinol Lett.

[30] Cui Y, Zhang H, Ji M, Jia M, Chen H, Yang J, Duan M (2014). Hydrogen-rich saline attenuates neuronal ischemia—reperfusion injury by protecting mitochondrial function in rats. J Surg Res.

[31] Nakao A, Kaczorowski DJ, Wang Y, Cardinal JS, Buchholz BM, Sugimoto R, Tobita K, Lee S, Toyoda Y, Billiar TR, McCurry KR (2010). Amelioration of rat cardiac cold ischemia/reperfusion injury with inhaled hydrogen or carbon monoxide, or both. J Heart Lung Transplant.

[32] Yin J, Tu C, Zhao J, Ou D, Chen G, Liu Y, Xiao X (2013). Exogenous hydrogen sulfide protects against global cerebral ischemia/reperfusion injury via its anti-oxidative, anti-inflammatory and anti-apoptotic effects in rats. Brain Res.

[33] Lee JC, Kim IH, Park JH, Ahn JH, Cho JH, Cho GS, Tae HJ, Chen BH, Yan BC, Yoo KY, Choi JH, Lee CH, Hwang IK, Cho JH, Kwon YG, Kim YM, Won MH (2015). Ischemic preconditioning protects hippocampal pyramidal neurons from transient ischemic injury via the attenuation of oxidative damage through upregulating heme oxygenase-1. Free Radic Biol Med.

[34] Jiang Z, Li C, Manuel ML, Yuan S, Kevil CG, McCarter KD, Lu W, Sun H (2015). Role of hydrogen sulfide in early blood-brain barrier disruption following transient focal cerebral ischemia. PLoS One.

[35] Chan SY, Loscalzo J (2010). MicroRNA-210 A unique and pleiotropic hypoxamir. Cell Cycle.

[36] Devlin C, Greco S, Martelli F, Ivan M (2011). miR-210: More than a silent player in hypoxia. IUBMB Life.

[37] Merlo A, de Quiros SB, Secades P, Zambrano I, Balbin M, Astudillo A, Scola B, Aristegui M, Suarez C, Chiara MD (2012). Identification of a signaling axis HIF-1alpha/microRNA-210/ISCU independent of SDH mutation that defines a subgroup of head and neck paragangliomas. J Clin Endocrinol Metab.

[38] Mei Y, Bian C, Li J, Du Z, Zhou H, Yang Z, Zhao RC (2013). miR-21 modulates the ERK-MAPK signaling pathway by regulating SPRY2 expression during human mesenchymal stem cell differentiation. J Cell Biochem.

[39] Liu P, Liang H, Xia Q, Li P, Kong H, Lei P, Wang S, Tu Z (2013). Resveratrol induces apoptosis of pancreatic cancers cells by inhibiting miR-21 regulation of BCL-2 expression. Clin Transl Oncol.

[40] Harms H, Reimnitz P, Bohner G, Werich T, Klingebiel R, Meisel C, Meisel A (2011). Influence of stroke localization on autonomic activation, immunodepression, and post-stroke infection. Cerebrovasc Dis.

[41] Miko I, Nemeth N, Sajtos E, Brath E, Peto K, Furka A, Szabo G, Kiss F, Imre S, Furka I (2010). Splenic function and red blood cell deformability: the beneficial effects of spleen autotransplantation in animal experiments. Clin Hemorheol Microcirc.

[42] Yang R, Qu C, Zhou Y, Konkel JE, Shi S, Liu Y, Chen C, Liu D, Chen Y, Zandi E, Chen W, Zhou Y, Shi S (2015). Hydrogen sulfide promotes Tet1-and Tet2-mediated Foxp3 demethylation to drive regulatory T cell differentiation and maintain immune homeostasis. Immunity.

[43] Wang Y, Zhou H, Shen Y, Wang Y, Wu W, Liu H, Yuan Z, Xu Y, Hu Y, Cao J (2015). Impairment of dendritic cell function and induction of CD4(+)CD25(+)Foxp3(+) T cells by excretory-secretory products: a potential mechanism of immune evasion adopted by *Echinococcus granulosus*. BMC Immunol.

[44] Assadiasl S, Ahmadpoor P, Nafar M, Lessan Pezeshki M, Parvin M, Shahlaee A, Sepanjnia A, Nicknam MH, Amirzargar A (2014). Regulatory T cell subtypes and TGF-beta1 gene expression in chronic allograft dysfunction. Iran J Immunol.

[45] Su Y, Fan W, Ma Z, Wen X, Wang W, Wu Q, Huang H (2014). Taurine improves functional and histological outcomes and reduces inflammation in traumatic brain injury. Neuroscience.

[46] Yang L, Zhang J, Yan C, Zhou J, Lin Q, Wang W, Zhang K, Yang G, Bian X, Zeng A (2012). sirt1 regulates cd40 expression induced by tnf-alpha via nf-kb pathway in endothelial cells. Cell Physiol Biochem.

[47] Ha TY (2011). The role of microRNAs in regulatory T cells and in the immune response. Immune Netw.

[48] Fayyad-Kazan H, Rouas R, Fayya-Kazan M, Badran R, Ei Zein N, Lewalle P, Najar M, Hamade E, Jebbawi F, Merimi M, Romero P, Burny A, Badran B, Martiat P (2012). MicroRNA profile of circulating CD4 positive regulatoryT cells in human adults and impact of differentially expressed microRNAs on expression of two genes essential to their function. J Biol Chem.

